# Important Announcement

**Published:** 1897-04

**Authors:** 


### IMPORTANT ANNOUNCEMENT.

THE JOURNAL is happy to have the privilege of announcing that before October 1, 1897, there will be issued by one of America's leading veterinarians a complete work on veterinary sanitary medicine, including meat- and milk-inspection. There will be about forty of the various infectious and contagious diseases taken up, involving the equine, bovine, ovine, porcine, canine, and feline species of animals, and reference to the leading scourges of fowls. This book will be broad, comprehensive, and up to date in every line of this work, and will be a complete guide and text-book in the field of veterinary sanitary medicine.

